# Impact of Previous Local Treatment for Brain Metastases on Response to Molecular Targeted Therapy in BRAF-Mutant Melanoma Brain Metastasis: A Systematic Review and Meta-Analysis

**DOI:** 10.3389/fonc.2022.704890

**Published:** 2022-06-24

**Authors:** Guixiang Liao, Yuxiang Fu, Sumbal Arooj, Muhammad Khan, Xianming Li, Maosheng Yan, Zihuang Li, Hongli Yang, Tao Zheng, Ruilian Xu

**Affiliations:** ^1^ Department of Radiation Oncology, Shenzhen People’s Hospital, The First Affiliated Hospital of Southern University of Science and Technology, Shenzhen, China; ^2^ Department of Biochemistry and Molecular Biology, University of Sialkot, Sialkot, Pakistan; ^3^ Department of Oncology, First Affiliated Hospital of Anhui Medical University, Hefei, China

**Keywords:** MEK inhibitors, BRAF inhibitors, previous therapy, prognosis, melanoma brain metastasis

## Abstract

**Background:**

Melanoma brain metastases (BMs) are associated with poor prognosis and are the main cause of mortality in melanoma patients. BRAF inhibitors have shown intracranial activity in both treatment-naïve and previously treated BM patients. We aimed to investigate if there was any difference in response of BRAF inhibitors in these two cohorts.

**Materials and Methods:**

Electronic database search included PubMed, Medline, and Cochrane library until March 2021 for studies with desired comparative outcomes. Outcomes of interest that were obtained for meta-analysis included intracranial response rate as the primary outcome and survival and safety outcomes as the secondary outcomes. Review Manager version 5.4 was used for data analysis.

**Results:**

Three studies comprising 410 BRAF-mutated melanoma patients with BMs were included according to eligibility criteria. The comparative cohort included patients with treatment-naïve BMs (TN cohort; *n* = 255) and those who had progressive disease after receiving local brain treatment for BMs (PT cohort; *n* = 155). Meta-analysis revealed that BRAF inhibitors (vemurafenib and dabrafenib) and BRAF/MEK inhibitor combination (dabrafenib and trametinib) induced significantly higher intracranial disease control (OR 0.58 [95% CI: 0.34, 0.97], *p* = 0.04) and a trend toward improved progression-free survival (PFS) (HR 1.22 [95% CI: 0.98, 1.52], *p* = 0.08) in the PT cohort as compared to the TN cohort. Overall survival was not significantly different between the cohorts (HR 1.16 [95% CI: 0.89, 1.51], *p* = 0.28). Subgroup analysis revealed that PFS was significantly improved (HR 1.67 [95% CI: 1.06, 2.62], *p* = 0.03), and a trend toward improved OS (HR 1.62 [95% CI: 0.95, 2.75], *p* = 0.08) was achieved in patients receiving BRAF/MEK inhibitor combination and patients with BRAFv600K mutation receiving dabrafenib alone. No increase in overall adverse events (AEs), grade 3/4 AEs, and severe adverse events (SAEs) was observed between the cohorts.

**Conclusions:**

BRAF inhibitors (plus MEK inhibitor) may achieve better intracranial disease stability in BRAF-mutant melanoma patients who have received previous local treatment for BMs.

**Systematic Review Registration:**

https://www.crd.york.ac.uk/prospero/), identifier CRD42020185984.

## Introduction

Melanoma is the fifth leading cause of cancer according to 2021 estimates of the American Cancer Society. An estimated 106,110 new cases of melanoma were diagnosed and 7,850 deaths from melanoma occurred in the United States in 2021 (1, 2). Melanoma represents the third most common cancer type (10%) that metastasizes to the brain after lung (50%) and breast cancer (20%) (3, 4). Approximately 20% of melanoma patients present with brain metastases (BMs) at diagnosis and approximately 10% to 44% develop BMs during the disease course (1–4). The risk of developing BMs is increased up to 80% in patients with metastatic melanoma (5). Patients with melanoma brain metastases (MBMs) have a poor prognosis with a median survival of 3.8 to 7.69 months (5–7).

Management of MBM involves surgery, whole brain radiotherapy (WBRT), and/or stereotactic radiosurgery (SRS) for BMs. WBRT is considered radioresistant with a poor outcome and SRS has been increasingly applied, which provides better local control (73%–90%) and median survival (5.3–10.5 months) as compared to WBRT alone. Systemic therapies offered include chemotherapy, molecular targeted therapy, and immunotherapy (8–10). Targeted therapy mainly involves BRAF inhibitors aimed at BRAF mutation, which is present in approximately 50% of metastatic melanoma (9, 10). BRAF mutation is reported with two types of substitutions: substitution of valine with glutamate at position 600 (Val600Glu/BRAFV600E) and with lysine (Val600Lys/BRAFv600K), which occurs in 70%–95% and 5%–30% of the cases, respectively (11–14). BRAF inhibitors, such as vemurafenib, dabrafenib, and encorafenib, and MEK inhibitors, such as selumetinib, trametinib, cobimetinib, and binimetinib, which target the mitogen-activated protein kinase (MAPK) pathway, have shown superior efficacy compared to chemotherapy in melanoma (15–19). Moreover, a combination of BRAF and MEK inhibitors has demonstrated greater efficacy than each agent alone (20–22). More importantly, these agents have also shown intracranial activity in BRAF-mutated melanoma patients with BMs (23–37). Several case reports, retrospective studies, and trial studies not only have unraveled vemurafenib’s protective effect against brain metastatic spread but also have shown intracranial response and safety (23–32). Similarly, dabrafenib has also demonstrated intracranial response in phase 1/2 trials in melanoma patients with BRAF (V600E/G/L) mutations (33, 34). Moreover, combinations, such as vemurafenib/cobimetinib, vemurafenib/trametinib, and encorafenib/binimetinib, have also demonstrated safety and intracranial activity (35, 37).

Several trials have demonstrated that BRAF inhibitors can induce intracranial responses in treatment-naïve and previously treated BMs derived from BRAF-mutant melanoma alike (32, 34, 35). Here, we attempt to systematically review and analyze the efficacy and safety outcomes as reported in these trials.

## Methods and Materials

PRISMA (Preferred Reporting Items for Systematic Reviews and Meta-Analyses) guidelines were followed in this review (38). A protocol of this study is registered on PROSPERO (https://www.crd.york.ac.uk/prospero/): CRD42020185984.

### Eligibility Criteria

The study population comprised BRAF-mutant melanoma patients with untreated BMs (control group) or progressive BMs after previous local treatments (experimental group) receiving BRAF inhibitors and/or MEK inhibitors. Outcomes of interest included efficacy and safety results such as intracranial response rate, progression-free survival (PFS), overall survival (OS), and treatment-related adverse events (AEs). Only clinical trials (randomized/non-randomized) with prospective design and comparative outcomes were considered for inclusion. Language was restricted to English.

### Information Sources

Explored databases included PubMed, Medline, and Cochrane library that were searched until April 10, 2021. Several search terms relevant to the eligibility criteria were employed accordingly. References of the relevant articles were further elaborated for potential studies.

### Study Selection

Retrieved studies were organized and screened for duplication and eligibility. Two reviewers (SA and MK) independently carried out the evaluation and selection of eligible studies. Disagreements were resolved after consulting with a third reviewer (GL).

### Data Extraction

A modified form of “The Cochrane Collaboration Data Collection form—RCTs and non-RCTs” was used for data extraction. Attributes of the studies included design, first author, publication year, number of participants, treatment regimens, and main efficacy and safety outcomes for the overall study group. Baseline characteristics of the patients included age, sex, performance status (Eastern Cooperative Oncology Group), BRAF mutation type, number of BMs, level of lactate dehydrogenase (LDH), and previous therapy. Furthermore, outcomes of interest (intracranial response rate, survival, and safety) for treatment differences were also extracted.

### Quality Assessment

Quality assessment was carried out with the checklist developed by the National Institute for Health and Care Excellence (NICE) (39). The checklist comprised eight questions that addressed various aspects of clinical case series.

### Measurement of Treatment Effect and Data Synthesis

The number of events for objective response rate (ORR) and disease control rate (DCR) at the intracranial and extracranial level were recorded from the studies to obtain odds ratio. Likewise, AEs were also recorded to obtain odds ratio. Hazard ratios for the survival outcomes (PFS and OS) were extracted from the Kaplan–Meier curves using the Digital Equalizer and methods for incorporating summary time-to-event data into the meta-analysis according to Tierney et al. (40, 41). The acquired odds ratios or hazard ratios were pooled using the software “RevMan 5.4 software” (42, 43). The Mantel–Haenszel method or inverse variance statistical method was applied for pooling obtained odds ratios and hazard ratios using the fixed effects analysis model, respectively (44). Heterogeneity was assessed using the chi-squared test and *I* ^2^ value. Heterogeneity was graded as low, moderate, and high according to *I* ^2^ values of 25%, 50%, and 75%, respectively (45). A random effects analysis model was used in case of moderate heterogeneity (≥50%). Significance level was set at *p* < 0.05.

## Results

**Figure 1:**
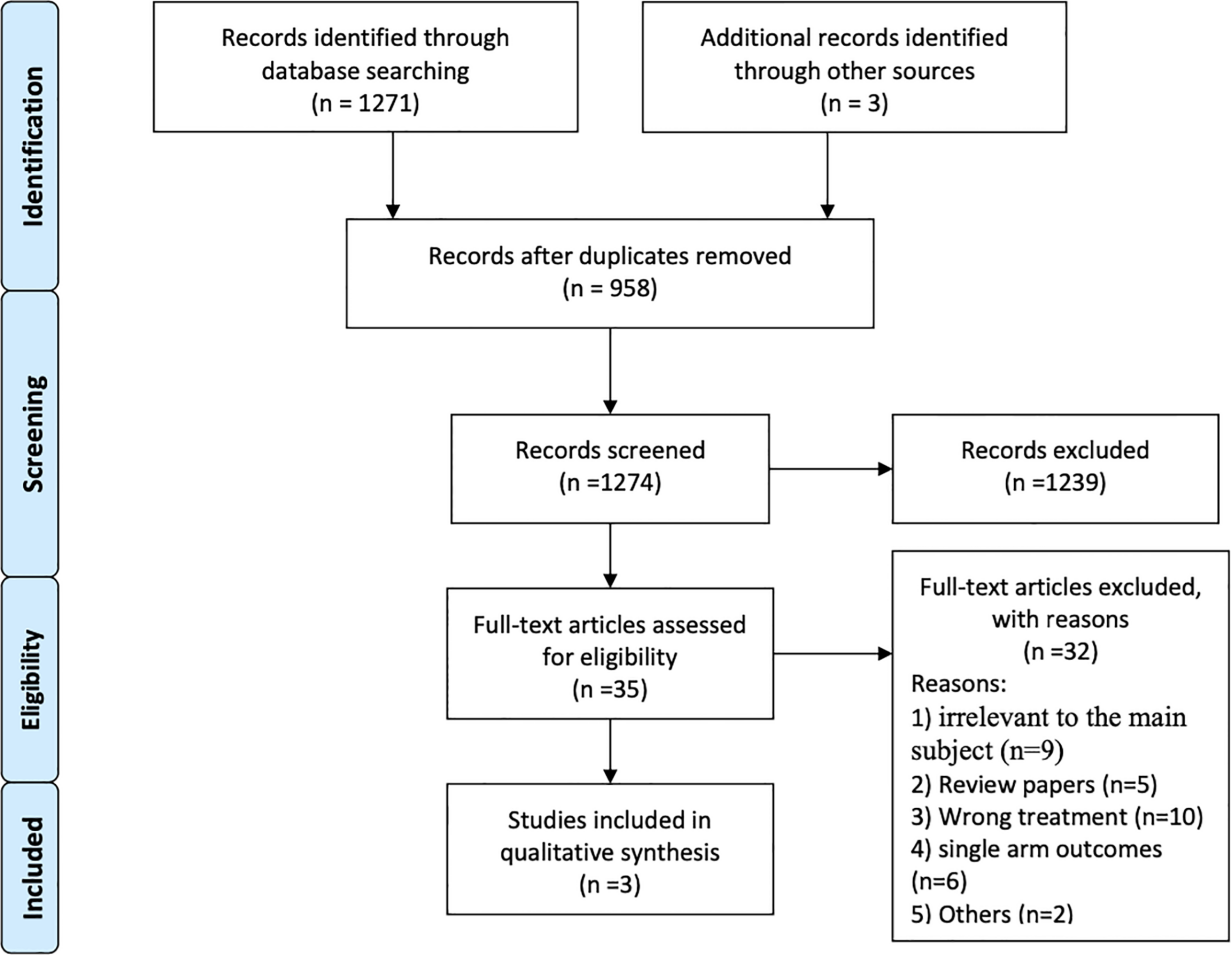
Flow diagram of research strategy and study selection.

A total of 1,271 articles were identified *via* an initial electronic database search. After exclusion for duplicity and eligibility, 35 articles were examined for detailed evaluation. Another 32 articles were excluded due to different reasons and the remaining 3 articles were finally selected for inclusion in this systematic review and meta-analysis (32, 34, 35). The search and selection process is detailed in **Figure 1**. All the three included studies were multicenter, open label, non-randomized, phase 2 clinical trials (32, 34, 35). Altogether, data of 410 BRAF-mutant melanoma brain metastatic patients treated with BRAF inhibitors (vemurafenib and dabrafenib) and/or MEK inhibitor (trametinib) were provided for analysis. Studies included mainly comprised two cohorts: patients with no local treatment administered to the brain (*n* = 255) and patients who had disease progression after having received local treatment to the brain (*n* = 155) in the form of surgery and/or radiation therapy (32, 34, 35). BMs were asymptomatic in two of the three trials (34, 35). General characteristics are illustrated in **Table 1**.

**Table 1:** General characteristics of the included clinical trials.

Study	Designation	Year	Sponsors	Participants	Mutation	Drug and Dosage	Primary endpoint	Follow-up (median)
Long, et al. (BREAK-MB) (34)	Multicenter, open-label, non-randomized, phase 2 trial (NCT01266967)	2012	GlaxoSmithKline	172	Val600Glu (BRAFV600E) or Val600Lys (BRAFV600K)	Dabrafenib 150 mg twice a day	Intracranial response	4 months*
McArthur, et al. (32)	Multicenter, open-label, non-randomized, phase 2 trial (NCT01378975)	2017	Hoffmann-La Roche.	146	BRAFV600	Vemurafenib 960 mg twice a day	Intracranial OR in cohort A	9.6 months
Davies, et al. (COMBI-MB) (35)	Multicenter, open-label, non-randomized, phase 2 trial (NCT02039947)	2017	Novartis (Novartis Pharmaceuticals)	92	BRAF V600E	Dabrafenib 150 mg twice daily plus trametinib 2 mg once daily	Intracranial response in cohort A	8.5 months

NCT, national clinical trial number; OR, objective response.

*At least 4 months.

Participants were predominantly male (*n* = 260) rather than female (*n* = 150). The majority of the patients (*n* = 374) were mainly BRAFV600E (Val600Glu); however, the study by Long et al. also had an additional comparative cohort (*n* = 33) with BRAFV600K (Val600Lys). The study by Long et al. provided data separately for Val600Glu and Val600Lys patients (35). Patients were evenly distributed according to age (*p* = 0.85), gender (*p* = 0.64), performance status (ECOG: 0 = 223, *p* = 0.92; 1 = 185, *p* = 0.98), BRAF genotype (*p* = 0.49), and lactate dehydrogenase level (elevated = 204, *p* = 0.29). Patients with single BM were predominant in the treatment-naïve cohort (TN) (OR 1.96 [95% CI: 1.27, 3.02], *p* = 0.002), while the previously treated cohort (PT) contained more patients with 2–4 BMs than the TN cohort (OR 0.63 [95% CI: 0.42, 0.96], *p* = 0.03). Moreover, the PT cohort was also more exposed to previous therapy than the TN cohort (OR 0.36 [95% CI: 0.23, 0.55], *p* < 0.00001). Baseline characteristics of the patients are outlined in **Table 2**. Quality assessment according to the eight items of the checklist are described in **Supplementary Table 1**. All clinical trials obtained a score of 7 out of 8 points.

**Table 2:** Baseline characteristics of study participants.

Studies	BREAK-MB		McArthur, et al. (2017) (32)		COMBI-MB		This study					
Cohorts	TN	PT	TN	PT	TN	PT	TN	PT	Total	OR (95% CI)	*I* ^2^ (%)	Significance
Characteristics												
No. of patients	89	83	90	56	76	16	255	155	410			
Age	52 (43–63)	53 (44–62)	55.5 (26–28)	52.5 (28–83)	52 (23–84)	54.5 (36–84)	–			−0.40 (−4.43, 3.62)*	0	*p* = 0.85
Sex												
Male	65	55	56	34	40	10	161	99	260	1.11 (0.72, 1.71)	0	*p* = 0.64
Female	24	28	34	22	36	6	94	56	150	0.90 (0.59, 1.39)	0	*p* = 0.64
ECOG												
0	48	51	42	21	50	11	140	83	223	0.98 (0.64, 1.49)	9	*p* = 0.92
1	41	32	47	35	25	5	113	72	185	1.00 (0.66, 1.51)	19	*p* = 0.98
2					1	0	1	0	1			
BRAF genotype												
BRAFV600E	74	65	90	56	73	16	237	137	374	1.29 (0.62, 2.69)	0	*p* = 0.49
BRAFV600K	15	18			3		18	18	36	0.77 (0.37, 1.60)	0	*p* = 0.49
Target BMs												
1	41	30	40	11	41	7	122	48	170	1.96 (1.27, 3.02)	23	*p* = 0.002
2–4	40	39	37	35	31	9	108	83	191	0.63 (0.42, 0.96)	33	*p* = 0.03
>4	8	14	13	10	4		25	24	49	0.66 (0.36, 1.22)	0	*p* = 0.19
Elevated LDH level	49	44	51	29	28	3	128	76	204	1.25 (0.82, 1.91)	0	*p* = 0.29
Previous therapy	30	53	18	22	17	5	65	80	145	0.36 (0.23, 0.55)	0	*p* < 0.00001

ECOG, Eastern Cooperative Oncology Group; TN, local treatment naïve cohort; PT, previous local treatment cohort; BM, brain metastases; LDH, lactate dehydrogenase.

*Mean difference.

### Intracranial Response Rate

**Figure 2:**
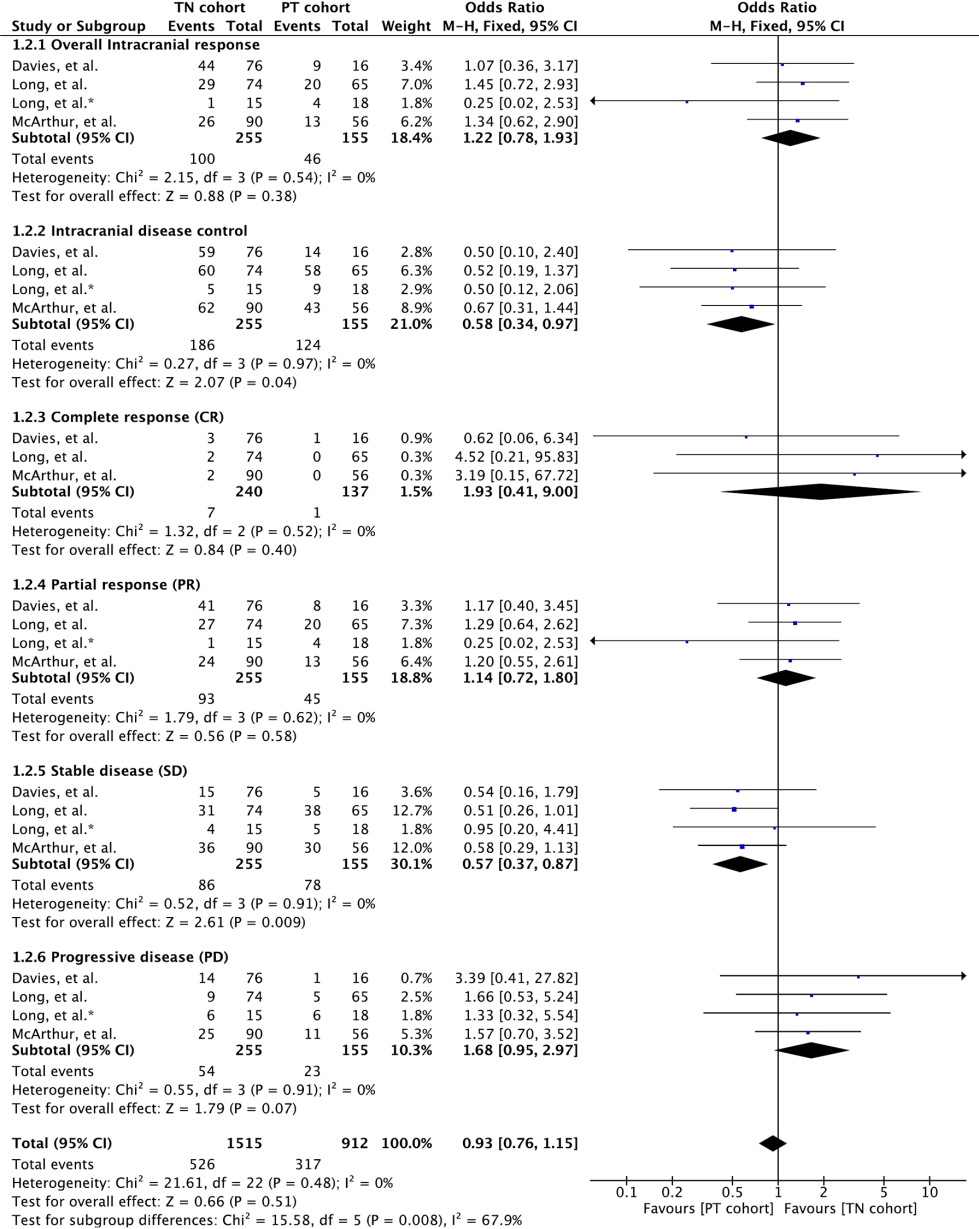
Forest plot of meta-analysis of intracranial response rate comparison between treatment-naïve (TN) and previously treated brain metastases (PT) cohorts in BRAF-mutant melanoma patients with brain metastases receiving BRAF/MEK inhibitors.

Intracranial response rate was reported in all three clinical trials involving 410 patients (32, 34, 35). All four parameters of intracranial response, namely, complete response (CR), partial response (PR), stable disease (SD), and progressive disease (PD), were reported across studies, and these were analyzed separately. Meta-analysis of overall intracranial response rate, defined as the combined number of patients achieving complete and partial response, revealed no significant difference between the cohorts (OR 1.22 [95% CI: 0.78, 1.93], *p* = 0*.38*) (**Figure 2**). Nonetheless, overall disease control, defined as the combined number of patients achieving CR, PR, and SD, was significantly higher in patients with previously treated BMs (OR 0.58 [95% CI: 0.34, 0.97], *p* = 0.04) (**Figure 2**).

Separate analysis of each intracranial response parameters showed that complete response rate was higher in treatment-naïve patients, although not significant (OR 1.93 [95% CI: 0.41, 9.00], *p* = 0.40). No significant difference between the cohorts was seen with respect to PR as well (OR 1.14 [95% CI: 0.72, 1.80], *p* = 0.58). Nonetheless, patients receiving previous local treatment achieved significantly higher disease stability compared to treatment-naïve patients (OR 0.57 [95% CI: 0.37, 0.87], *p* = 0.009). Moreover, treatment-naïve patients tended to experience high disease progression (OR 1.68 [95% CI: 0.95, 2.97], *p* = 0.07) (**Figure 2**).

Moreover, duration of intracranial response was reported in all three studies (32, 34, 35) (**Supplementary Table 2**). Vemurafenib-induced median duration of intracranial response was 4.7 months (range; 2.7–24.2) in the TN cohort and 6.6 months (range; 1.9–22.0) in the PT cohort (32). Median duration of intracranial response with dabrafenib was also slightly higher in the PT cohort compared to that in the TN cohort (BRAFV600E group: 28.1 [95% CI: 20.1, 28.1] versus 20.1 [95% CI: 12.1, NR]/BRAFV600K group: 16.6 [95% CI: NR, NR] versus 12.4 [95% CI: NR, NR]) (34). A similar trend was also observed in patients receiving dabrafenib plus trametinib (7.3 [95% CI: 3.6, 12.6] versus 6.5 [95% CI: 4.9, 10.3]) (35).

### Extracranial Response Rate

**Figure 3:**
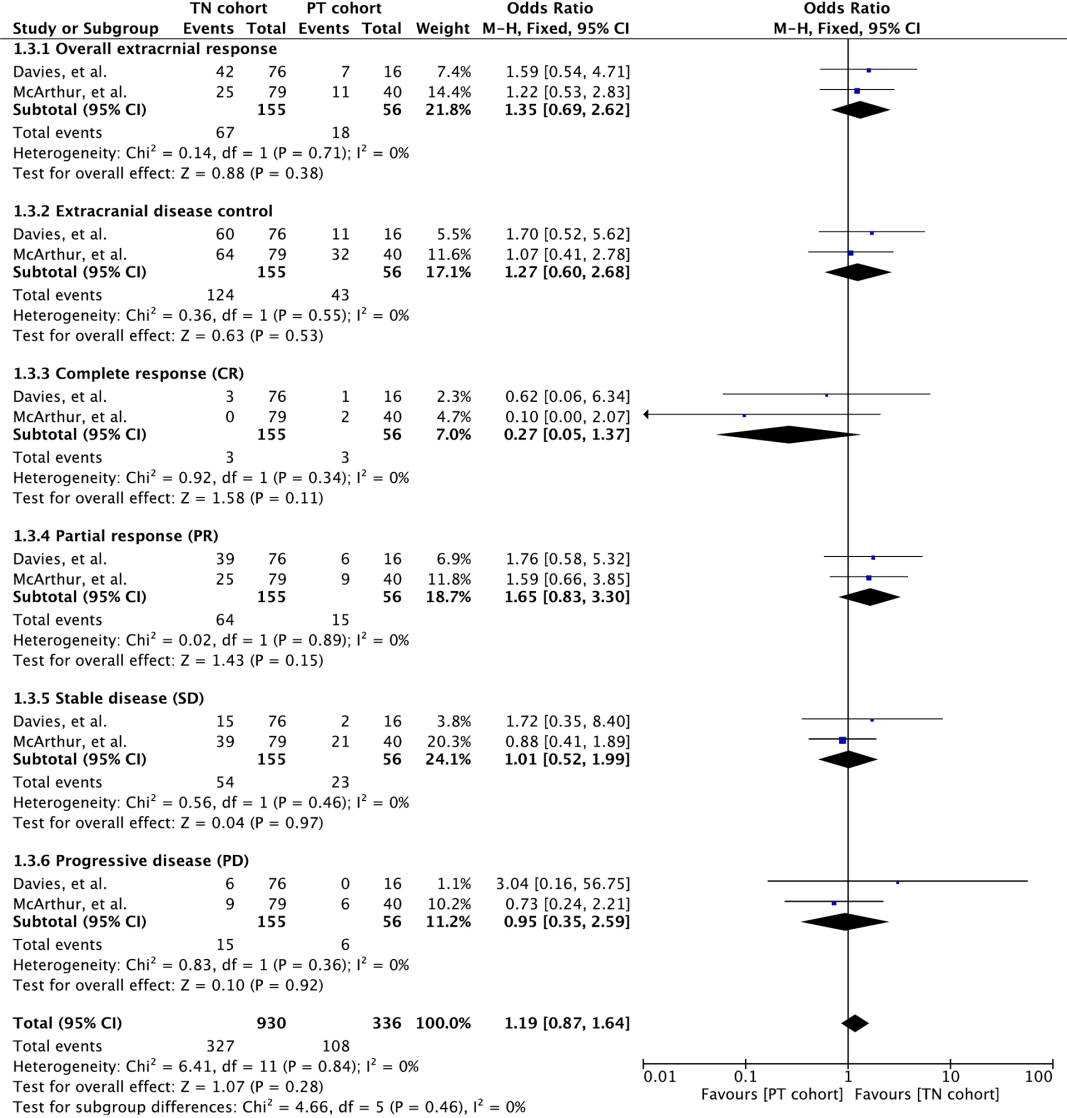
Forest plot of meta-analysis of extracranial response rate comparison between treatment-naïve (TN) and previously treated brain metastases (PT) cohorts in BRAF-mutant melanoma patients with brain metastases receiving BRAF/MEK inhibitors.

Extracranial response rate was reported in only two clinical trials involving 211 patients (32, 35). These studies involved vemurafenib and the combination of dabrafenib and trametinib. Meta-analysis of overall extracranial response rate revealed no significant difference between the cohorts (OR 1.35 [95% CI: 0.69, 2.62], *p* = 0.38). Overall disease control was also insignificant for cohort difference (OR 1.27 [95% CI: 0.60, 2.68], *p* = 0.53). None of the response parameters were significant for cohort difference (CR: OR 0.27 [95% CI: 0.05, 1.37], *p* = 0.11; PR: OR 1.65 [95% CI: 0.83, 3.30], *p* = 0*.15*; SD: OR 1.01 [95% CI: 0.52, 1.99], *p* = 0.97; PD: OR 0.95 [95% CI: 0.35, 2.59], *p* = 0.92). Although meta-outcome was insignificant, dabrafenib and trametinib demonstrated stronger extracranial activity in treatment-naïve BM cohort, as shown in **Figure 3**.

Duration of extracranial response was reported in two studies wherein vemurafenib and dabrafenib plus trametinib were administered to patients (32, 35) (**Supplementary Table 2**). The vemurafenib-induced median duration of intracranial response was 5.6 months (range: 1.8–25.6) in the TN cohort and 10.7 months (range: 1.8–23.1) in the PT cohort (32). A trend towards better extracranial response rate was also observed in patients receiving dabrafenib plus trametinib (NE [95% CI: NE, NE] versus 10.2 [95% CI: 5.8, NE]) (35).

### Overall Response Rate

**Figure 4:**
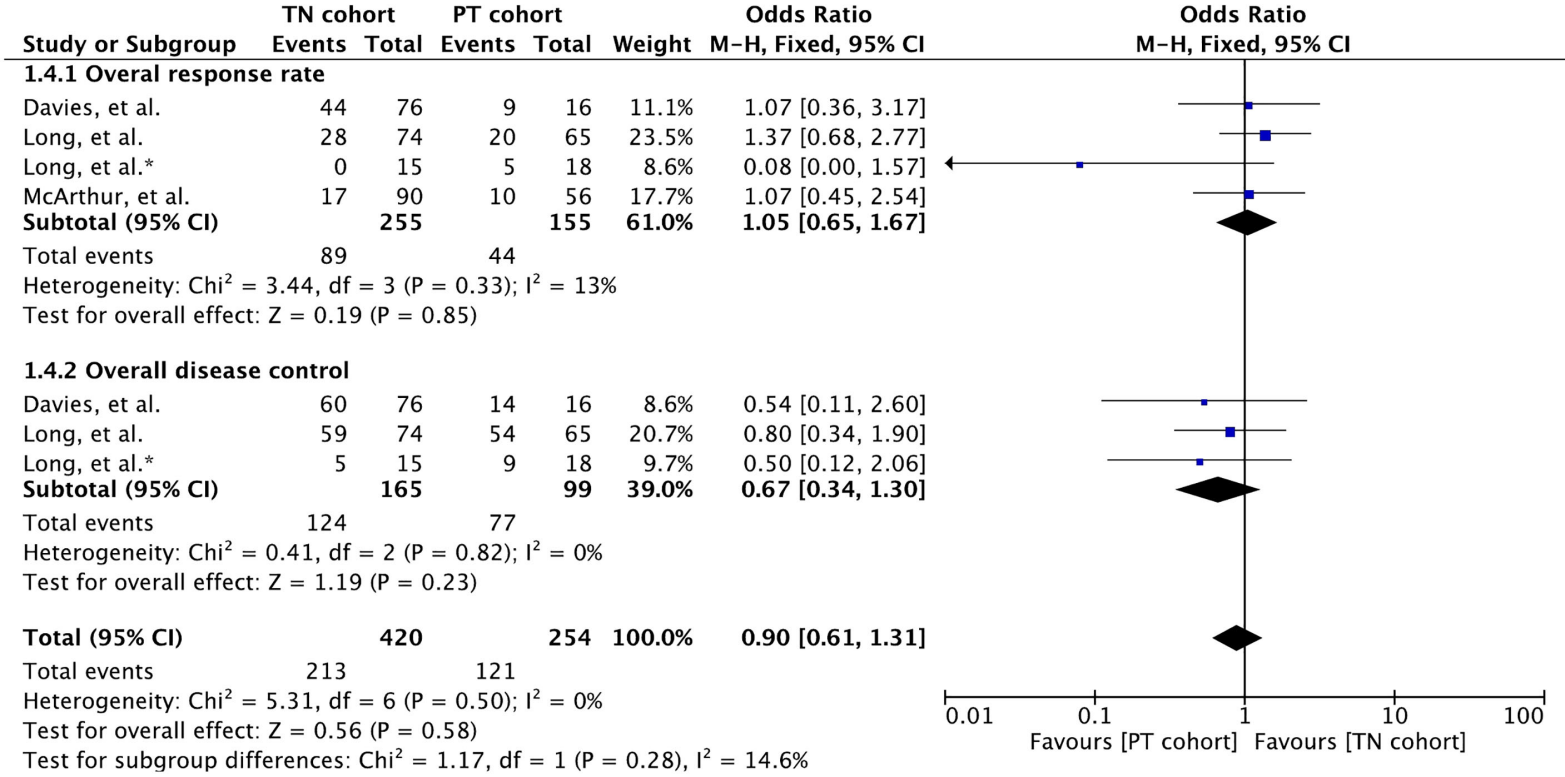
Forest plot of meta-analysis of overall response rate (both intracranial and extracranial) comparison between treatment-naïve (TN) and previously treated brain metastases (PT) cohorts in BRAF-mutant melanoma patients with brain metastases receiving BRAF/MEK inhibitors. * Indicates separate comparison for participants with BRAFV600K (Val600Lys) mutation provided by (34).

All three studies reported overall response rate involving 410 patients (32, 34, 35). Overall response rate was defined as the proportion of patients with a best response of CR or PR when both extracranial and intracranial disease were assessed in them. Meta-analysis of overall response rate showed no significant difference between the cohorts (OR 1.05 [95% CI: 0.65, 1.65], *p* = 0.85) (**Figure 4**). Likewise, no difference was noted in the overall disease control (OR 0.67 [95% CI: 0.34, 1.30], *p* = 0.23) reported in two studies involving 264 patients (34, 35).

Duration of overall response (intracranial and extracranial response) was reported in only one study with patients receiving dabrafenib plus trametinib (35) (**Supplementary Table 2**). Median duration of intracranial response was slightly higher in the PT cohort compared to that in the TN cohort (12.5 [95% CI: 5.3, NE] versus 6.5 [95% CI: 4.9, 10.3] (35).

### Progression-Free Survival

**Figure 5:**
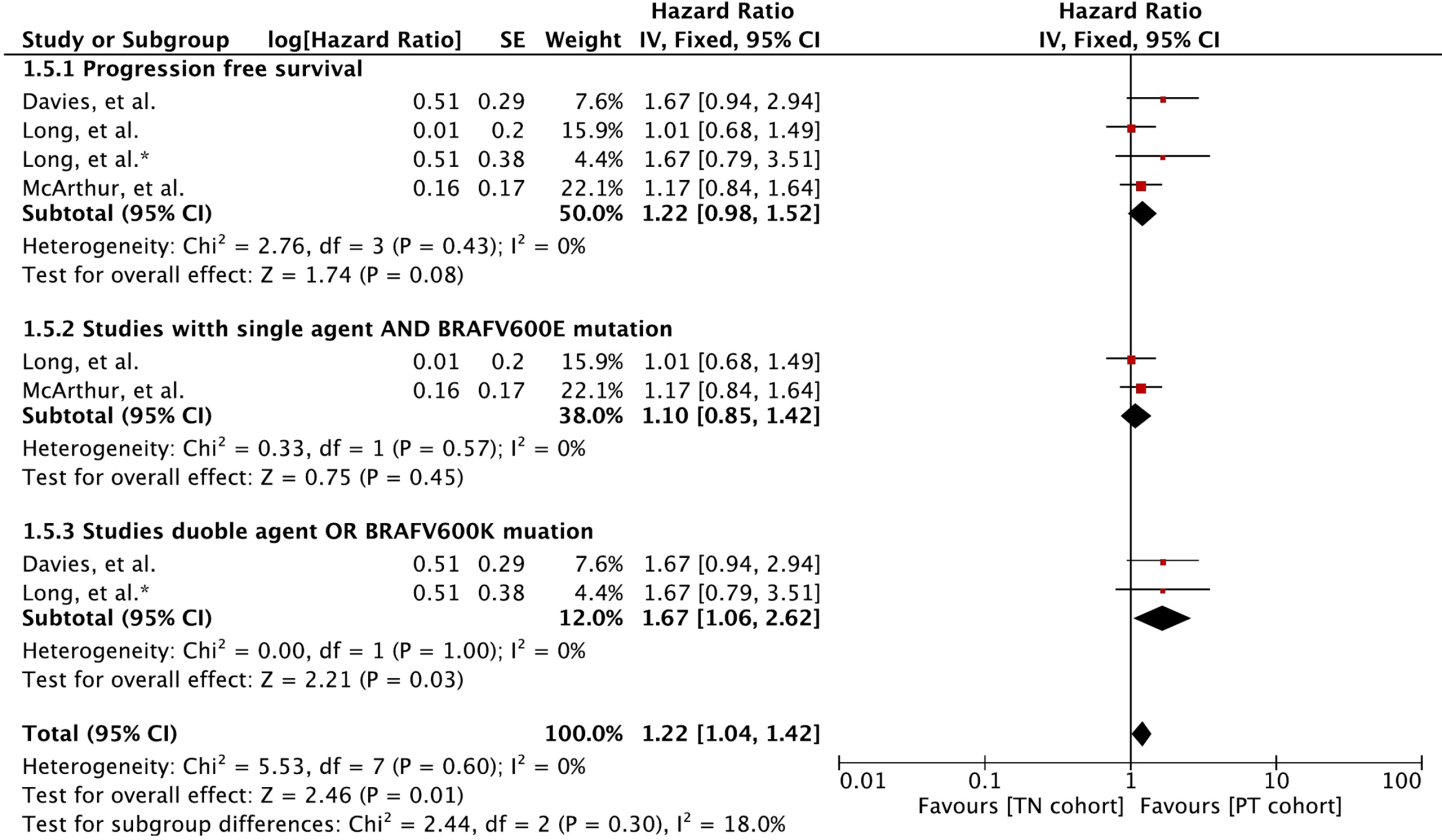
Forest plot of meta-analysis of progression-free survival comparison between treatment-naïve (TN) and previously treated brain metastases (PT) cohorts in BRAF-mutant melanoma patients with brain metastases receiving BRAF/MEK inhibitors. * Indicates separate comparison for participants with BRAFV600K (Val600Lys) mutation provided by (34).

Meta-analysis of PFS involving 410 participants resulted in slightly better PFS for MBM patients with previously treated BMs (**Figure 5**). A hazard ratio of 1.22 (95% CI: [0.98, 1.52], *p* = 0.08) was revealed for the cohort difference.

Subgroup analysis revealed that MBM patients with BRAFV600E (Val600Glu) mutation and treated with a single agent had no difference in PFS (HR 1.10 [95% CI: 0.85, 1.42], *p* = 0.45). The PFS was mainly derived with BRAF and MEK inhibitor combination or patients with BRAFV600K (Val600Lys) (HR 1.67 [95% CI: 1.06, 2.62], *p* = 0.03).

### Overall Survival

**Figure 6:**
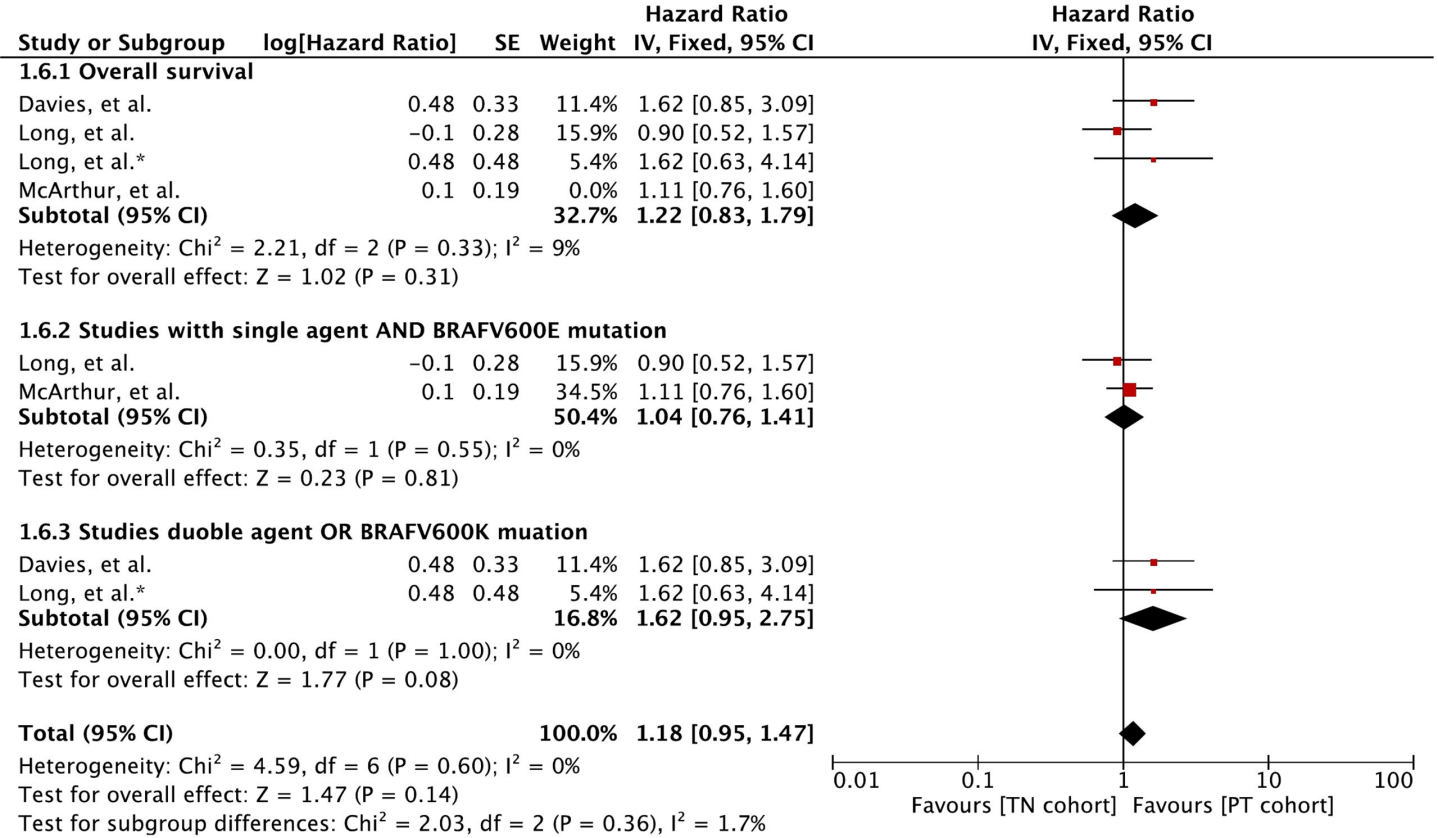
Forest plot of meta-analysis of overall survival comparison between treatment-naïve (TN) and previously treated brain metastases (PT) cohorts in BRAF-mutant melanoma patients with brain metastases receiving BRAF/MEK inhibitors. * Indicates separate comparison for participants with BRAFV600K (Val600Lys) mutation provided by (34).

Meta-analysis of OS involving 410 participants showed no difference between the cohorts (**Figure 6**). A hazard ratio of 1.16 (95% CI: [0.89, 1.51], *p* = 0.28) was revealed for the cohort difference.

Subgroup analysis revealed that MBM patients with BRAFV600E (Val600Glu) mutation and treated with a single agent had no difference in OS (HR 1.04 [95% CI: 0.76, 1.41], *p* = 0.81). The OS derived with BRAF and MEK inhibitor combination or patients with BRAFV600K (Val600Lys) was slightly better in the previously treated cohort (HR 1.62 [95% CI: 0.95, 2.75], *p* = 0.08).

### Adverse Events

**Figure 7:**
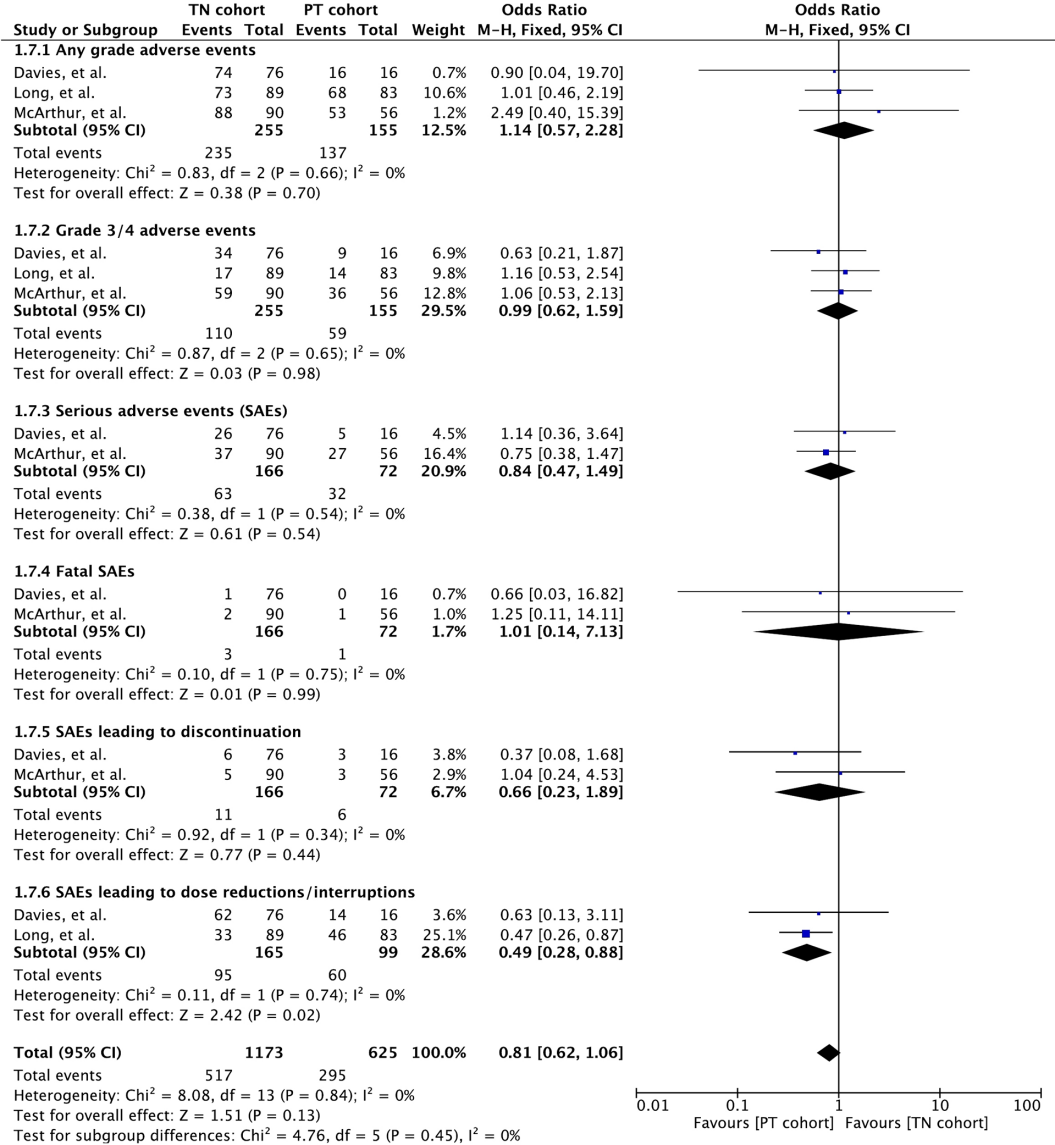
Forest plot of meta-analysis of adverse event rate comparison between treatment-naïve (TN) and previously treated brain metastases (PT) cohorts in BRAF-mutant melanoma patients with brain metastases receiving BRAF/MEK inhibitors.

All three included studies reported safety of these drugs in detail (32, 34, 35). AEs were assessed according to the National Cancer Institute Common Terminology Criteria for Adverse Events (version 4.0) grading system. Meta-analysis of any grade toxicity revealed no significant difference between the cohorts (OR 1.14 [95% CI: 0.57, 2.28], *p* = 0.70) (**Figure 7**). Likewise, there was no difference in grade 3/4 toxicity between the cohorts (OR 0.99 [95% CI: 0.62, 1.59], *p* = 0.98).

Most common AEs as reported varied slightly from study to study. The most common events triggered by vemurafenib included arthralgia (54/146; 37%), rash (47/146; 32%), hyperkeratosis/fatigue (41/146; 28%), photosensitivity reaction (35/146; 24%), prolonged ECG QT (30/146; 21%), nausea/alopecia (29/146; 20% each), skin papilloma (27/146;19%), squamous cell carcinoma of the skin (17/146;12%), and keratoacanthoma (15/146; 10%) (32). Pyrexia (44/172; 26%) of any grade and cutaneous squamous-cell carcinoma (11/146; 6%) were the most common AEs associated with dabrafenib (34). AEs induced by dabrafenib plus trametinib included pyrexia (*n* = 34/125; 54%), headache (*n* = 46/125; 37%), asthenia/diarrhea/nausea (*n* = 40/125; 32% each), and chills (*n* = 37/125; 30%) (35).

### Serious Adverse Events

Serious adverse events (SAEs) were reported in all three studies (32, 34, 35). Meta-analysis revealed no difference (OR 0.84 [95% CI: 0.47, 1.49], *p* = 0.54) between the cohorts based on data from two studies (*n* = 238) (32, 35) (**Figure 7**).

The study by Long et al. involving dabrafenib alone revealed overall SAEs for the entire study population (34). Overall, 51 (30%) patients experienced SAEs including pyrexia (10/172; 6%), intracranial hemorrhage (10/172; 6%), and squamous cell carcinoma (11/172; 6%). Vemurafenib-induced SAEs included squamous cell carcinoma of the skin (17/64; 12%), keratoacanthoma (15/64; 10%), and malignant melanoma (4/64; 3%) (32). The most common SAEs were pyrexia with dabrafenib (*n* = 8/124; 6%) and increase in ejection fraction with trametinib (*n* = 5/125; 4%) (35).

### Fatal SAEs

Meta-analysis of fatality caused by SAEs revealed no difference (OR 1.01 [95% CI: 0.14, 7.13], *p* = 0.99) between the cohorts based on data from the two studies (*n* = 238) (32, 35) (**Figure 7**). No treatment-related death was reported in the study by Long et al. (34). AEs causing death in patients receiving vemurafenib were pneumonia (2 deaths; one in each cohort) and glioma (1 death in cohort B) (32). Intracranial tumor hemorrhage was the cause of the only SAE-related death in the cohort A receiving dabrafenib plus trametinib (35).

### Discontinuation/Dose Interruptions

Discontinuation and dose interruptions of study treatment on account of SAEs were recorded in all the three studies (32, 34, 35). Based on the data from two studies (*n* = 238), discontinuation resulted in no significant difference between cohorts (OR 0.66 [95% CI: 0.23, 1.89], *p* = 0.44) (32, 35) (**Figure 7**). The study by Long et al. also mentioned discontinuation by 4 patients on account of treatment-related AEs (34).

Dose interruptions were required more in patients with previously treated BMs in two studies (*n* = 264) that involved dabrafenib and dabrafenib plus trametinib (OR 0.49 [95% CI: 0.28, 0.88], *p* = 0.02) (34, 35).

### Publication Bias

Publication bias was assessed using a funnel plot for all the main outcomes, including intracranial/extracranial response rate, survival outcomes, and AEs. All results were within the 95% CI, indicating no evidence of publication bias (**Supplementary Figures 1** **–** **6**).

## Discussion

Our results comprising 410 BRAF-mutated melanoma brain metastatic patients indicate that BRAF inhibitors (vemurafenib and dabrafenib) alone or in combination with MEK inhibitor (trametinib) are active and safe in BRAF-mutated melanoma with treatment-naïve or previously treated BMs. Even though the treatment-naïve cohort consisted of significantly more single BMs and fewer 2–4 BMs, intracranial DCR was significantly higher (*p* = 0.04) in previously treated brain metastatic patients as compared to treatment-naïve BMs. The main difference was observed in intracranial disease stability (high in previously treated BMs; *p* = 0.0009) and progression (high in treatment-naïve BMs; *p* = 0.07). Consequently, a slight trend towards better PFS (*p* = 0.08) was observed with no significant improvement in OS. Subgroup analysis revealed that a significant improvement in PFS (*p* = 0.03) and an almost significant OS (*p* = 0.08) could be achieved in previously treated BMs when analysis was restricted to patients receiving a combination of dabrafenib and trametinib and patients with BRAFV600K (Val600Lys) mutation.

Vemurafenib, dabrafenib, and dabrafenib plus trametinib have shown intracranial responses in previous studies in which participants were either untreated or heavily treated or were of the mixed type (23–37). For example, vemurafenib has previously shown intracranial responses in heavily pretreated MBMs. In a pilot study of vemurafenib, a partial response and stable disease in four patients was achieved in a total of five heavily pretreated MBM patients (29). Likewise, in a phase 2 trial of 24 pretreated MBM patients, both intracranial (*n* = 19; PR: 3, SD: 13) and extracranial responses (*n* = 21; PR: 13, SD: 6) were observed with 3.9 (95% CI: 3.0, 5.5) months of PFS and a median OS of 5.3 (95% CI: 3.9, 6.6) months (30). A separate trial of vemurafenib that included mixed BRAF-mutated MBM patients (treatment-naïve or previously treated BMs) also showed vemurafenib activity (*n* = 66; CR:1, PR:11, SD:34, and PD:10) with a PFS of 4.8 months (95% CI: 3.7, 5.7) and OS of 7.9 (95% CI: 5.9, 9.3) (31). A similar objective response and survival outcome (PFS and OS) for vemurafenib were demonstrated in both treatment-naïve and previously treated BMs in the study by McArthur et al., which was included in our meta-analysis (32). Previously, dabrafenib was only investigated in a small cohort of 10 BRAF-mutated melanoma patients with untreated BMs, which revealed excellent intracranial (*n* = 10; CR: 4, PR: 9) and extracranial (*n* = 10; PR: 9) activity, and yielded 4.2 months (95% CI: 3.3, 5.3) of PFS (33). BREAK MB trial reveals that like vemurafenib, dabrafenib is also active and safe in both cohorts of BMs. COMBI-MB trial revealed efficacy and safety of dabrafenib for the first time in a combination of trametinib in both cohorts. The combination has been previously proven to be more effective than vemurafenib and dabrafenib alone in metastatic melanoma without BMs (21, 46). Although the odds of intracranial response DCR were similar in the PT cohort, the combination of dabrafenib and trametinib was associated with slightly improved PFS and OS (35). In fact, extracranial ORR and DCR were also slightly favoring the treatment-naïve patients in their study (35). Nonetheless, no patient with progressive disease (0/16) and a small cohort may have caused improved PFS and OS (35). Therefore, this outcome needs to be validated in studies with larger cohorts. On the other hand, improved PFS and OS in BRAFV600K was in coherence with its intracranial efficacy in a cohort with previously treated BMs (34). Although overall response showed no difference between cohorts, extracranial activity alone was not reported in these studies. Nonetheless, duration of response was also longer in the PT cohort across all studies.

The activity and efficacy observed with BRAF/MEK inhibitors in both cohorts appeared to be superior and in slight contrast to the intracranial activity and survival benefit achieved with temozolomide in MBM patients (47). An objective response of 7% (8 patients) and stable disease of 29% (34 patients) was demonstrated for patients who had not received previous treatment for brain lesions with a median survival of 3.5 months. Response in previously treated BMs was slightly lower with temozolomide, revealing a partial response and 18% (6 patients) stable disease with a median survival of 2.2 months (47). Patients with previous radiotherapy have benefited in preclinical and clinical studies for immune checkpoint blockade (48, 49). In a secondary analysis of the KEYNOTE-001 phase 1 trial, the benefit for NSCLC patients with previous radiotherapy receiving pembrolizumab was evident in terms of PFS (HR 0.56 [95% CI: 0.34, 0.91], *p* = 0.019) and OS (HR 0.58 [95% CI: 0.36, 0.94], *p* = 0.*026*) (49). In a meta-analysis of BRAF-mutant MBM patients, those administered BRAF inhibitors concurrently or after SRS induction showed superior benefit than patients receiving prior SRS (HR 0.39 [95% CI: 0.24, 0.65], *p* = 0.0003) (50). Similarly, in a retrospective study, MBM patients receiving BRAF inhibitor after SRS were noted to have better survival compared to those receiving BRAF inhibitors concurrently (24 months vs. 10.1 months, *p* = 0.007) (51). Hence, it appears that patients with previous radiotherapy may be at an advantage for deriving better outcomes compared to treatment-naïve cancer patients. However, the underlying mechanism for this finding remains to be elucidated.

No increase in overall AEs, grade 3/4 AEs, and SAEs were reported between the cohorts that used a single agent or a combination of agents. Only increased dose reductions or interruptions were required with dabrafenib and/or trametinib. Our results were limited by several factors. The number of studies and total population of our study was small. In the study by Davies et al., the PT cohort had a small number of patients (*n* = 16) (35). All the trials lacked randomization and blinding, which may have rendered these studies prone to selection, performance, and detection biases (32, 34, 35). Moreover, agents used were different and patients had both types of BRAF mutation.

## Conclusions

Vemurafenib, dabrafenib, and dabrafenib plus trametinib demonstrate intracranial activity and safety in BRAF-mutated melanoma (BRAFV600E) patients with treatment-naïve or previously treated BMs. Intracranial disease control was significantly higher in patients with previously treated BMs. Moreover, patients with previously treated BMs had improved PFS and/or OS with dabrafenib plus trametinib compared to treatment-naïve patients. In BRAFV600K (Val600Lys) mutated melanoma, dabrafenib may improve PFS and OS in patients with previously treated BMs. Randomized controlled trials (RCTs) with larger cohorts would, however, be required to validate these outcomes accordingly.

## Data Availability Statement

The original contributions presented in the study are included in the article/**Supplementary Material**. Further inquiries can be directed to the corresponding authors.

## Author Contributions

All authors listed have made a substantial, direct, and intellectual contribution to the work and approved it for publication.

## Funding

This work was supported by the Natural Science Foundation of Shenzhen (No. JCYJ20170307095828424), Shenzhen Health and Family Planning System Research Project (No. SZBC2017024), and the Technical Research and Cultivation Project for the Youth of Shenzhen People’s Hospital (No. SYKYPY2019029).

## Conflict of Interest

The authors declare that the research was conducted in the absence of any commercial or financial relationships that could be construed as a potential conflict of interest.

## Publisher’s Note

All claims expressed in this article are solely those of the authors and do not necessarily represent those of their affiliated organizations, or those of the publisher, the editors and the reviewers. Any product that may be evaluated in this article, or claim that may be made by its manufacturer, is not guaranteed or endorsed by the publisher.
