# Supplementary material for: Impact of Previous Local Treatment for Brain Metastases on Response to Molecular Targeted Therapy in BRAF-Mutant Melanoma Brain Metastasis: A Systematic Review and Meta-Analysis
Source: Front Oncol. 2022 Jun 24;12:704890. doi: 10.3389/fonc.2022.704890 (PMC9263360; doi:10.3389/fonc.2022.704890)

**Impact of Previous Local Treatment for Brain Metastases on Response to Molecular Targeted Therapy in BRAF-Mutant Melanoma Brain Metastasis: A Systematic Review and Meta-Analysis**

**Supplementary Appendix**

This appendix has been provided by the authors to give readers additional information about their work.

| **Supplementary Table 1: Quality assessment checklist** | | | |
| --- | --- | --- | --- |
| **Quality assessment questionnaire** | **Long, et al. (2012).**  **(BREAK-MB)** | **McArthur, et al. (2017).** | **Davies, et al. (2017).**  **(COMBI-MB)** |
| **1. Case series collected in more than one centre, i.e. multi-centre study?** | √ | √ | √ |
| **2. Is the hypothesis/aim/objective of the study clearly described?** | √ | √ | √ |
| **3. Are the inclusion and exclusion criteria (case definition) clearly reported?** | √ | √ | √ |
| **4. Is there a clear definition of the outcomes reported?** | √ | √ | √ |
| **5. Were data collected prospectively?** | √ | √ | √ |
| **6. Is there an explicit statement that patients were recruited consecutively?** | X | X | X |
| **7. Are the main findings of the study clearly described?** | √ | √ | √ |
| **8. Are outcomes stratified? (e.g., by disease stage, abnormal test results, patient characteristics)** | √ | √ | √ |
| **Total score (__/8)**  (Yes=1; No=0) | 7/8 | 7/8 | 7/8 |

**Supplementary Table 1**

**Supplementary Table 2**

| **Supplementary Table 2: Duration of response** | | | | | | |
| --- | --- | --- | --- | --- | --- | --- |
| **Studies** | **BREAK-MB*** | | **McArthur, et al. (2017).** | | **COMBI-MB** | |
| cohorts | TN | PT | TN | PT | TN | PT |
| **Intracranial duration of response** | | | | | | |
| Median (95% CI), months | 20.1 (12.1-NR) | 28.1 (20.1-28.1) | 4.7 (rang; 2.7-24.2) | 6.6 (range; 1.9-22.0) | 6.5 (4.9-10.3) | 7.3 (3.6-12.6) |
|  | 12.4 (NR-NR)# | 16.6 (NR-NR)# |  |  |  |  |
| **Extracranial duration of response** | | | | | | |
| Median (95% CI), months |  |  | 5.6 (range; 1.8-25.6) | 10.7 (range; 1.8-23.1) | 10.2 (5.8-NE) | NE (NE-NE) |
| **Overall duration of response** | | | | | | |
| Median (95% CI), months |  |  |  |  | 6.5 (4.9-10.3) | 12.5 (5.3-NE) |
| Abbreviations: TN=local treatment naïve cohort; PT=previous local treatment cohort; NR=not reached; NE=not estimable; CI=confidence interval  # duration of response reported in BRAFV600K cohort  *Duration of response BREAK-MB was calculated in those patients with an intracranial partial or complete response. | | | | | | |

**Funnel plot of publication bias assessment**

**Supplementary Figure 1.** Funnel plot of publication bias assessment in intracranial response analysis. All studies are within the 95% confidence interval.


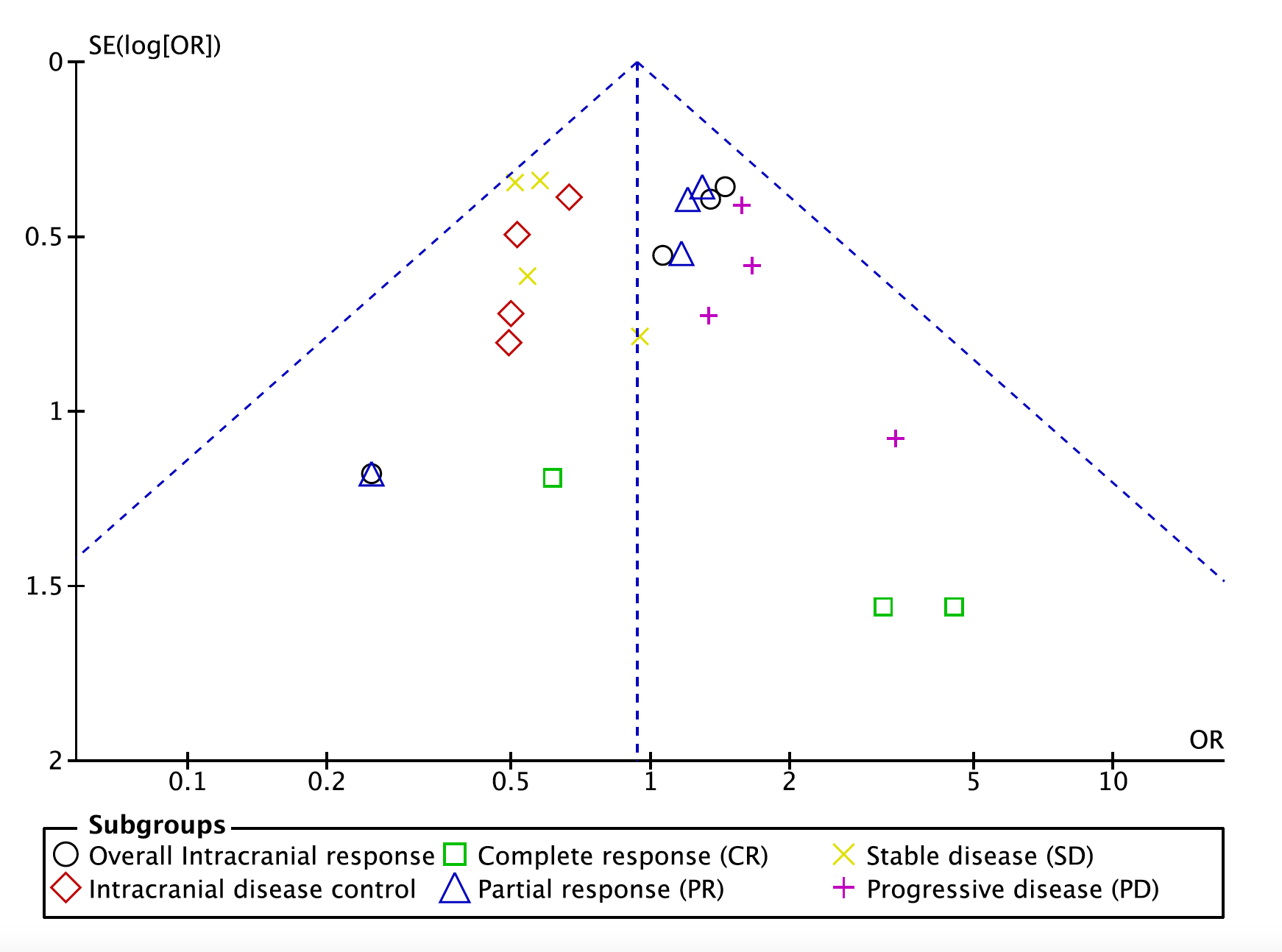


**Supplementary Figure 2.** Funnel plot of publication bias assessment in extracranial response analysis. All studies are within the 95% confidence interval.


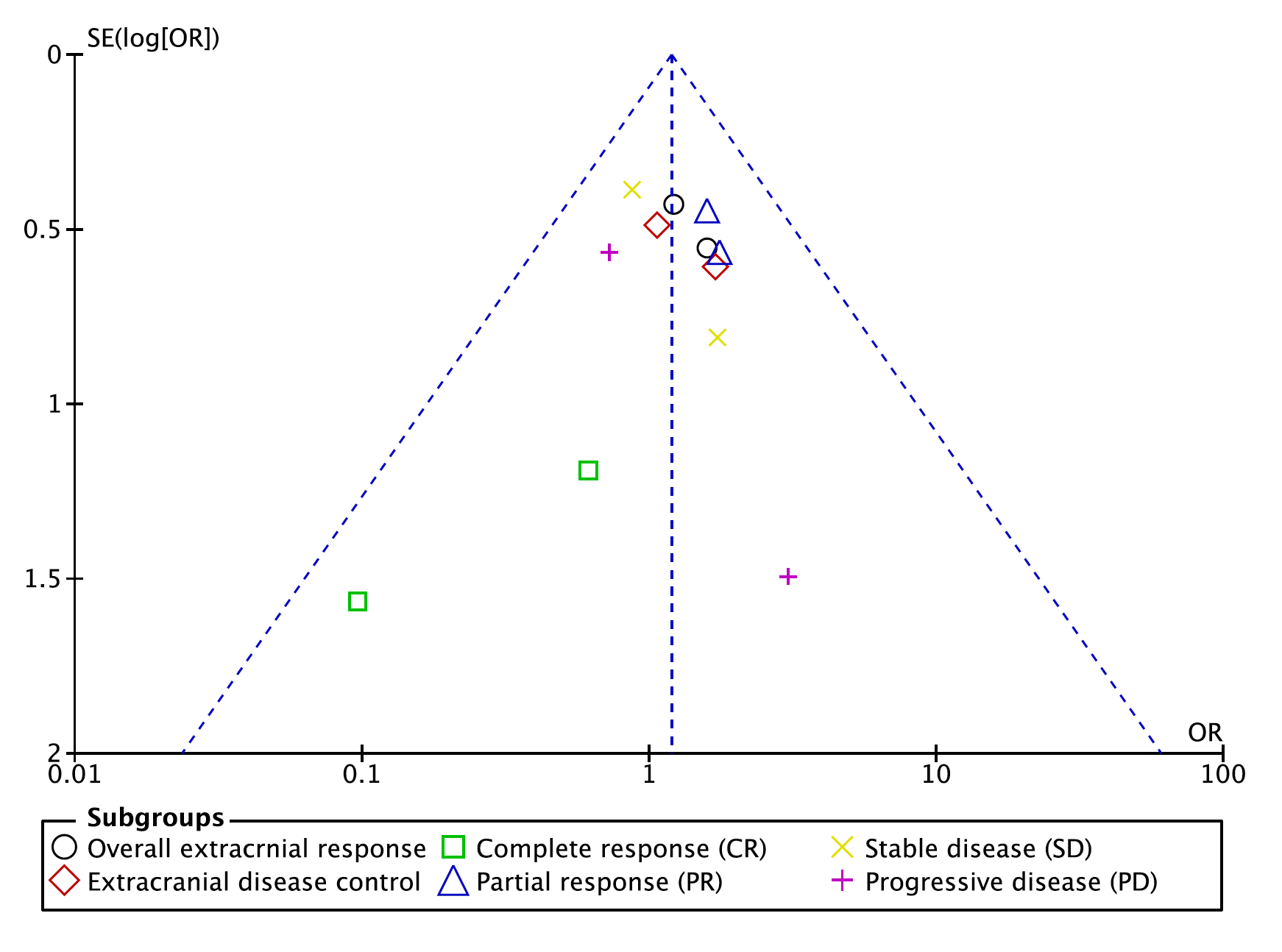


**Supplementary Figure 3.** Funnel plot of publication bias assessment in Extracranial response analysis. All studies are within the 95% confidence interval.


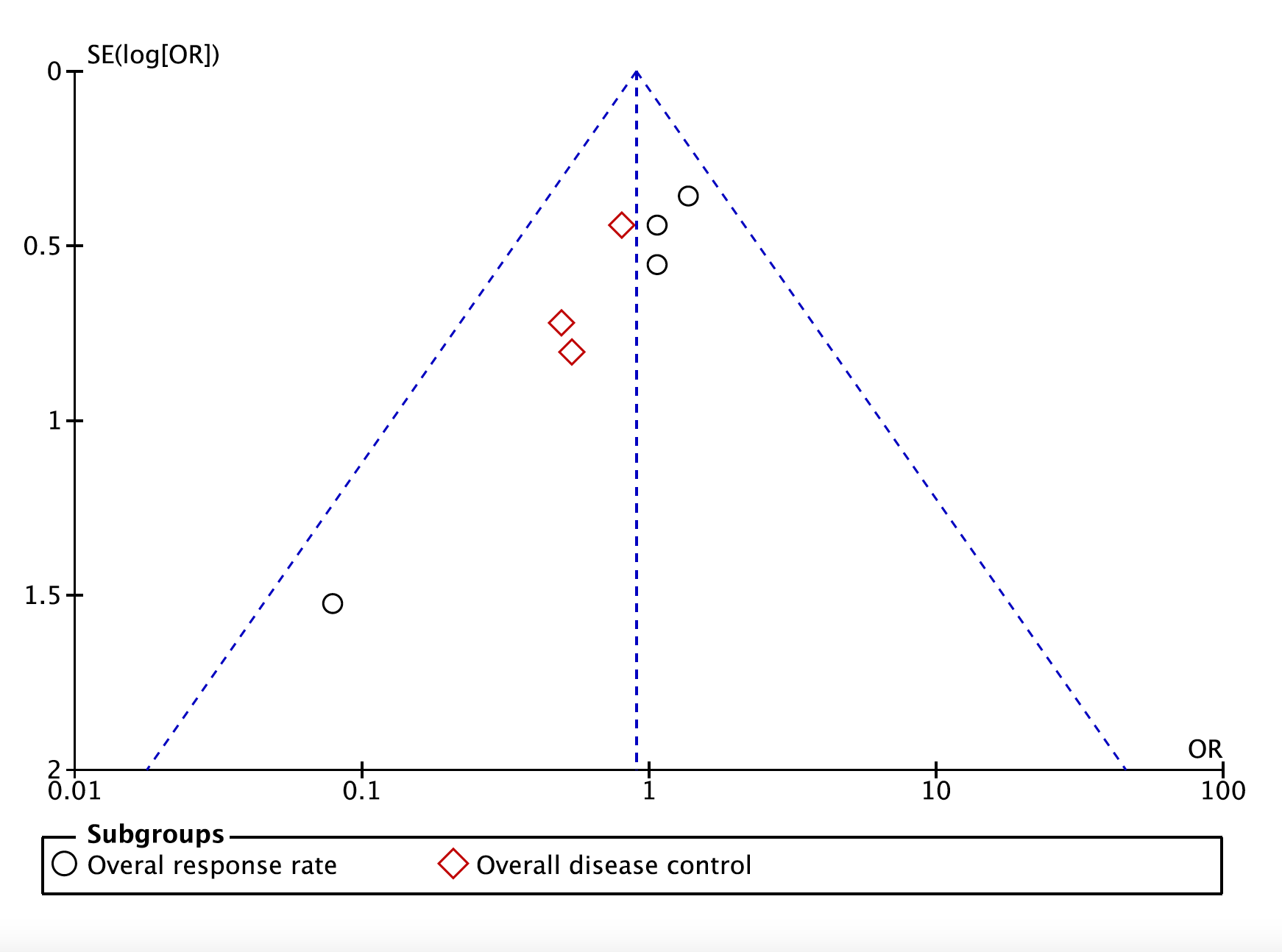


**Supplementary Figure 4.** Funnel plot of publication bias assessment in Progression free survival analysis. All studies are within the 95% confidence interval.


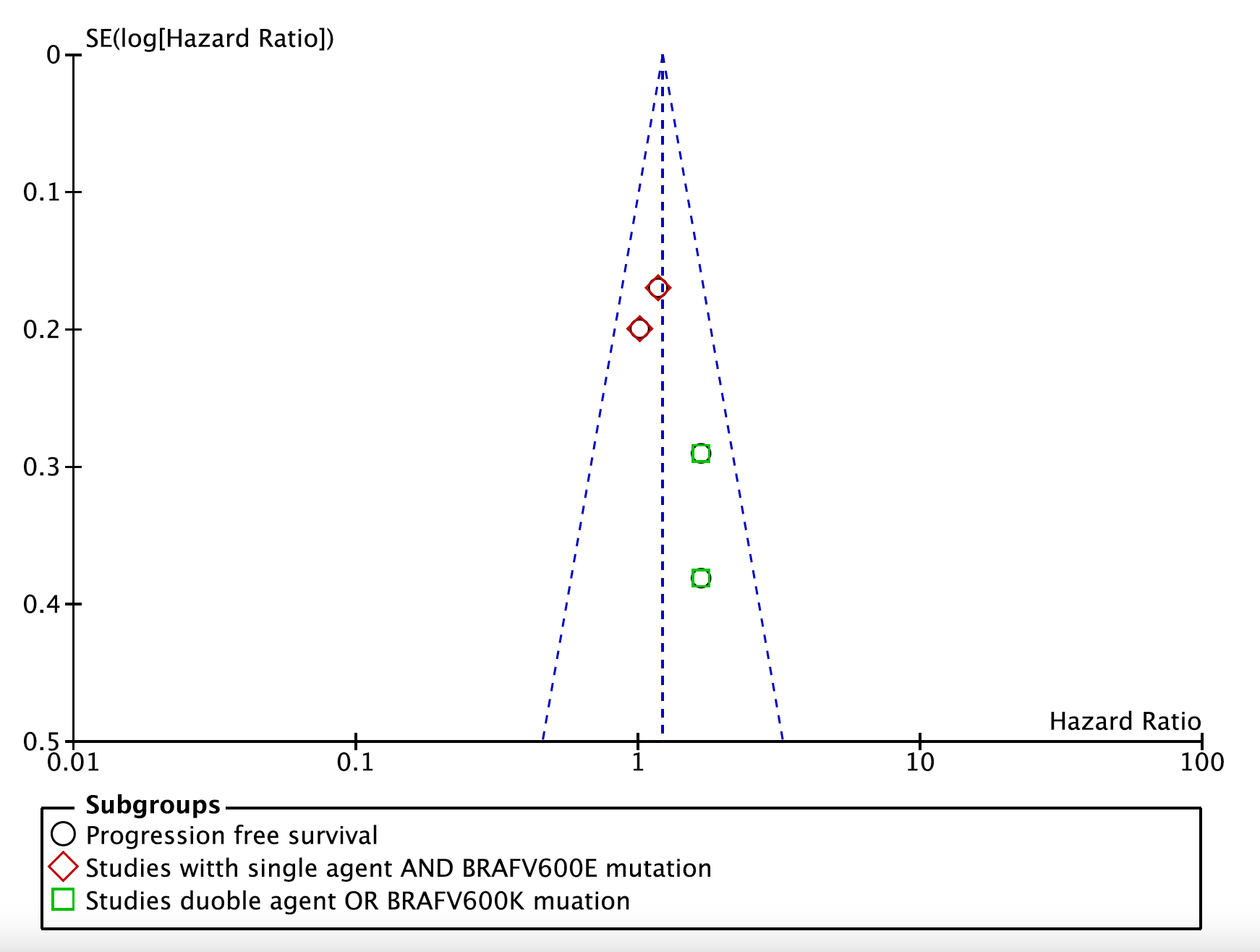


**Supplementary Figure 5.** Funnel plot of publication bias assessment in overall survival analysis. All studies are within the 95% confidence interval.


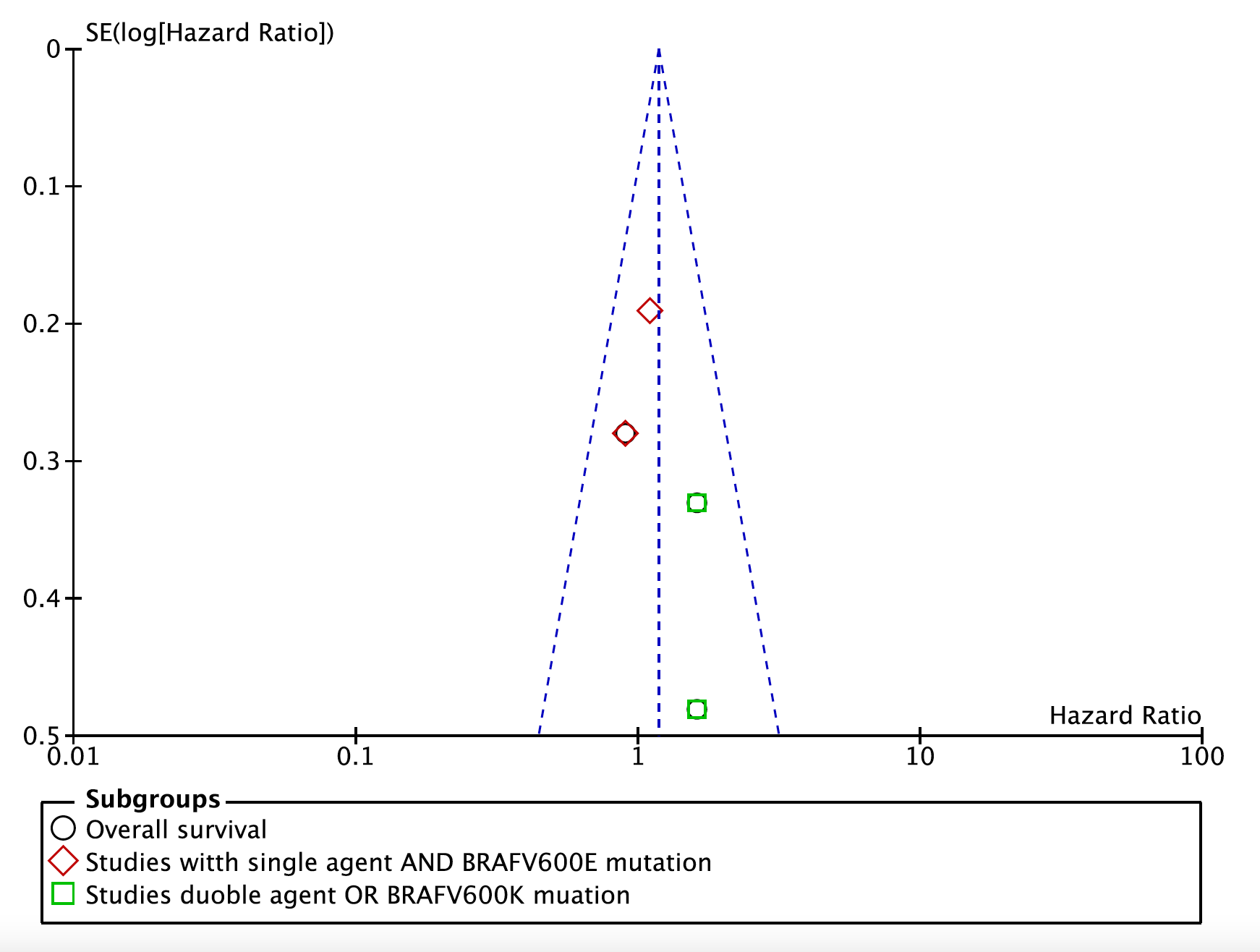


**Supplementary Figure 6.** Funnel plot of publication bias assessment in adverse events analysis. All studies are within the 95% confidence interval.


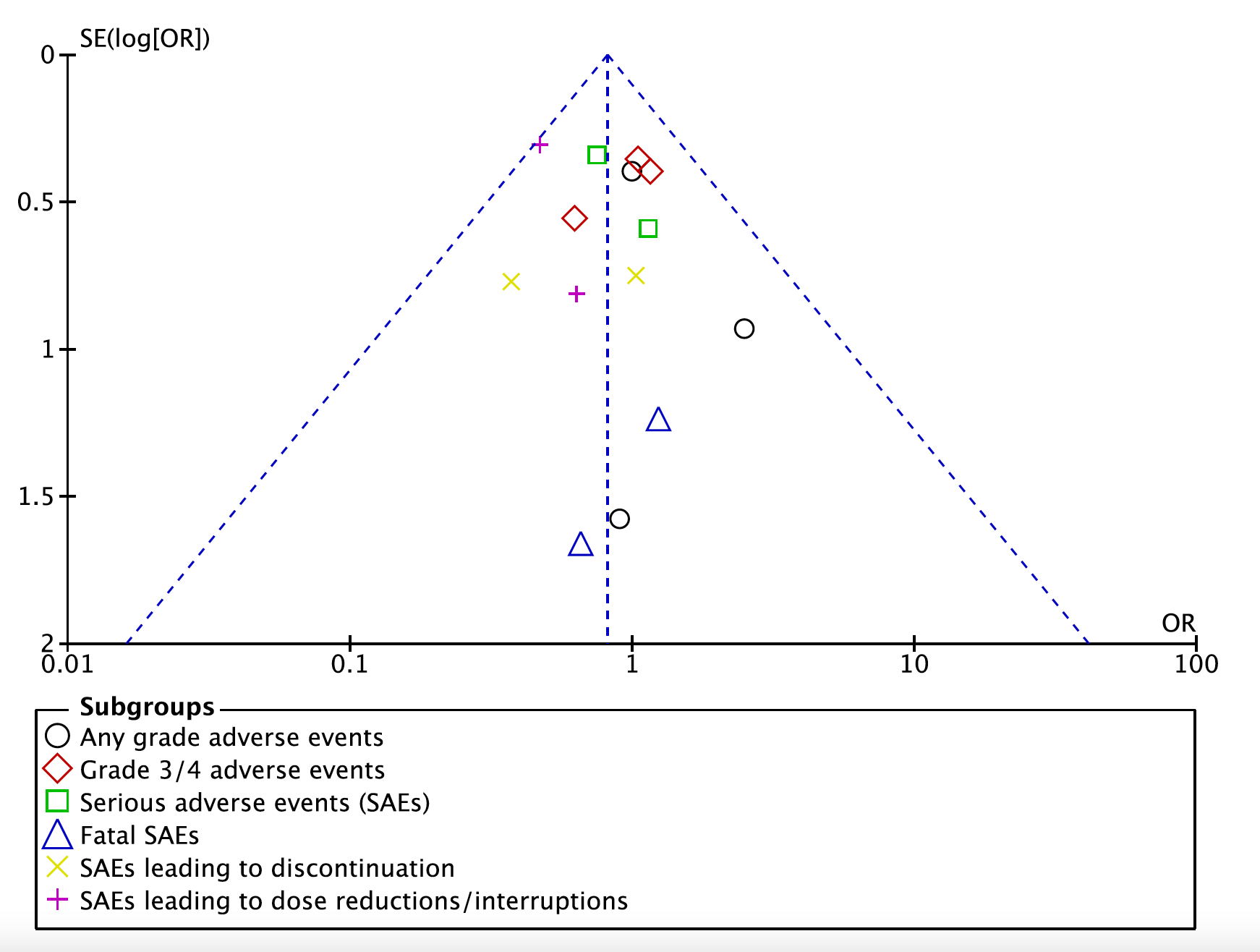

Supplement: Supplementary file 1 [file DataSheet_1.docx]
